# Survival benefit of adjuvant therapy completion with early initiation for patients with pancreatic ductal adenocarcinoma

**DOI:** 10.1002/ags3.12898

**Published:** 2024-12-25

**Authors:** Kenjiro Okada, Kenichiro Uemura, Tatsuaki Sumiyoshi, Ryuta Shintakuya, Kenta Baba, Takumi Harada, Yasutaka Ishii, Shiro Oka, Yoshiaki Murakami, Shinya Takahashi

**Affiliations:** ^1^ Department of Surgery Graduate School of Biomedical and Health Sciences, Hiroshima University Hiroshima Japan; ^2^ Department of Gastroenterology Graduate School of Biomedical and Health Sciences, Hiroshima University Hiroshima Japan; ^3^ Advanced Medicine of Hiroshima Memorial Hospital Hiroshima University Hospital Hiroshima Japan

**Keywords:** adjuvant chemotherapy, pancreatectomy, pancreatic diseases, pancreatic ductal carcinoma, postoperative complications

## Abstract

**Aim:**

To evaluate the prognostic effect of initiation timing and completion of adjuvant therapy in patients with pancreatic ductal adenocarcinoma.

**Methods:**

The medical records of patients with pancreatic ductal adenocarcinoma who underwent radical pancreatectomy between 2006 and 2022 at Hiroshima University were retrospectively reviewed. Patient characteristics, perioperative outcomes, clinicopathological factors, and survival rates were analyzed. Adjuvant indications were for all patients who had a good postoperative status as early as possible. Early initiation was defined as adjuvant initiation within 4 weeks after surgery, and completion was defined as a total of 6 months of administration.

**Results:**

In total, 444 (294, resectable; 150, borderline resectable or locally advanced) patients who received adjuvant therapy were enrolled in this study. The median time to adjuvant therapy initiation was 20 days. In total, 328 patients with early initiation had better overall survival than those with delayed initiation, and 409 patients with adjuvant completion had better survival rates than those with incompletion. Multivariate overall survival analysis demonstrated that early adjuvant therapy initiation and completion were independent prognostic factors for prolonged survival. In total, 310 adjuvant completions with early initiation resulted in a median survival period of 81.8 months. Multivariate analysis identified severe postoperative complication as an independent risk factor preventing adjuvant completion with early initiation.

**Conclusion:**

Adjuvant completion with early initiation may contribute to the improved survival of patients with pancreatic ductal adenocarcinoma. Preventing severe postoperative complications may facilitate adjuvant completion with early initiation.

## Graphical abstract

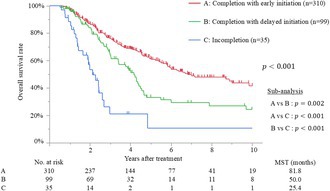

## 1 INTRODUCTION

Pancreatic ductal adenocarcinoma (PDAC) is one of the most lethal malignancies and the fourth most common cause of cancer‐related deaths in Western countries, with a 5‐year overall survival rate of 13%.^1^ Surgical resection is the only potentially curative treatment for PDAC, but survival rates after resection remain extremely poor. To improve the survival of patients with PDAC, some randomized studies have shown the survival benefits of adjuvant therapy after surgery. Adjuvant therapy is a strong prognostic factor for patients with resected PDAC and is a standard treatment following curative surgery.^2^, ^3^

However, the optimal time to initiate adjuvant therapy in patients with PDAC remains controversial. Some studies^4^, ^5^, ^6^, ^7^, ^8^, ^9^ evaluated patient prognosis stratified by various cut‐off timings of adjuvant initiation (8–12 weeks), and the results differed among the studies. Most of these reports focused on the timing of adjuvant initiation; however, it was unclear whether the patients completed adjuvant therapy. Adjuvant completion improves the survival of patients with PDAC, and it is necessary to verify which adjuvant initiation timing and completion contribute to improved survival. Therefore, this study aimed to evaluate the prognostic effect of adjuvant therapy initiation timing and completion in patients with PDAC.

## 2 METHODS

### 2.1 Study design

This retrospective cohort study was based on a prospectively maintained institutional database of all patients with PDAC who underwent potentially curative resection at the Department of Surgery, Hiroshima University Hospital, Hiroshima, Japan, between 2006 and 2022. In this study, we initiated adjuvant therapy as early as possible for patients with pathologically confirmed PDAC who recovered sufficiently after surgery. Adjuvant regimens were combined with gemcitabine plus S‐1 (GS) therapy^10^, ^11^, ^12^ or S‐1 monotherapy,^13^ and radiation therapy was not performed. The exclusion criteria were as follows: (1) patients who did not receive adjuvant therapy, (2) patients who had early recurrence or death within 6 months after surgery, and (3) patients lost to follow‐up within 6 months after surgery. We evaluated preoperative patient status, perioperative outcomes, adjuvant status, and survival. This study was approved by the Institutional Review Board of Hiroshima University and conducted in accordance with the principles of the Declaration of Helsinki (approval number: E‐2151).

### 2.2 Definition

This study focused on two factors of adjuvant therapy for patients with PDAC: (1) the initiation timing (early or delayed) and (2) completion status. Early initiation was defined as initiation within 4 weeks after surgery, whereas delayed initiation was defined as initiation beyond 4 weeks after surgery. The number of patients who received adjuvant therapy each week after surgery was also assessed. Completion of adjuvant therapy was defined as a total of 6 months administration after surgery. Tumor stage, lymph node metastasis, and final stage were classified based on the 8th edition of the International Union Against Cancer tumor‐node‐metastasis classification.^14^ All eligible patients had their resectability status assessed according to the National Comprehensive Cancer Network Pancreatic Cancer guidelines, version 2.2023.^15^ The incidence and severity of postoperative complications were graded according to the Clavien–Dindo classification system,^16^ and grade III or higher complications were considered severe complications. Overall survival was defined as the interval between treatment initiation and the date of death or last follow‐up visit.

### 2.3 Statistical analyses

Analyzed data were described using the medians and interquartile ranges (IQRs) for continuous variables and numbers for categorical variables. Categorical variables were compared using chi‐squared or Fisher's exact tests. Deaths from any cause were included in the survival analysis, and survival curves were plotted using the Kaplan–Meier method. Multivariate survival analyses were performed using the Cox proportional hazards model, and hazard ratios (HRs) and 95% confidence intervals (CIs) were reported. Risk factors were evaluated using univariate and multivariate logistic regression models, and odds ratios (ORs) and 95% CIs were reported. All tests were two‐sided, and *p* < 0.05 was considered significant. Statistical analyses were performed using JMP software (version 17.0; SAS Institute, Cary, NC, USA).

## 3 RESULTS

### 3.1 Patients

**FIGURE 1:**
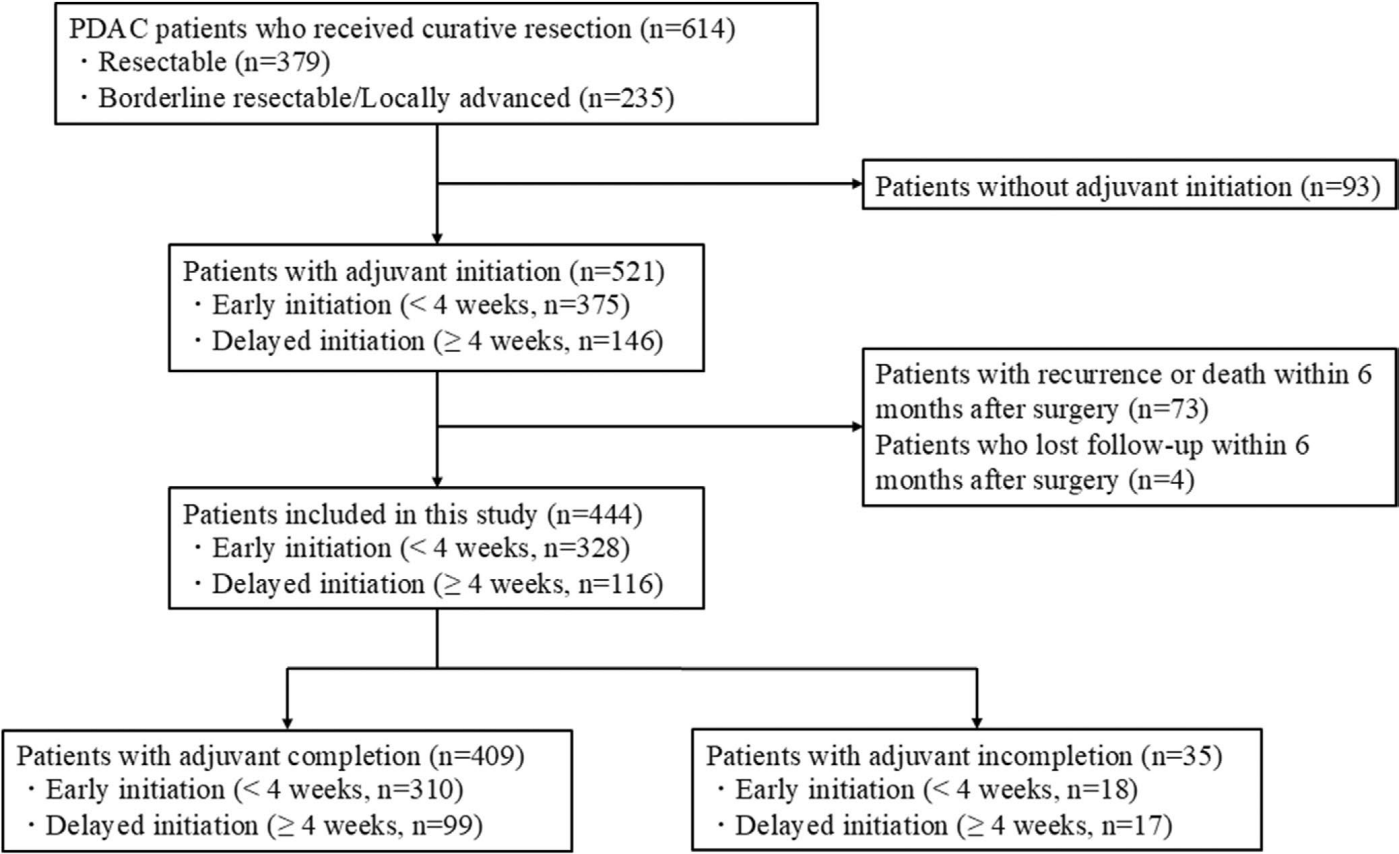
Patient flowchart.

The patient flow chart is shown in Figure 1. During the study period, 614 patients received curative resection for PDAC, including 379 resectable (R) and 235 borderline resectable (BR) or locally advanced (LA) cases. Of the 614 patients, 93 without adjuvant initiation after surgery were excluded. In the remaining 521 patients, 73 patients with recurrence or death within 6 months after surgery and four patients who were lost to follow‐up within 6 months after surgery were also excluded. The remaining 444 patients, including 294 R‐PDAC and 150 BR/LA‐PDAC cases, were included in this study; 311 patients were initially administrated combined GS therapy and 133 were administered S‐1 monotherapy.

The median time to initiation was 20 days (IQR; 15–29). The eligible 444 patients were classified into two groups: 328 patients (74%) with early initiation within 4 weeks after surgery and 116 (26%) with delayed initiation beyond 4 weeks after surgery. Of the 444 patients, 409 patients (92%), including 310 with early initiation and 99 with delayed initiation, achieved completion of adjuvant therapy, while 35 patients (8%) did not achieve completion. Patient demographics, surgical outcomes, and clinicopathological factors were shown in Table 1 and Table S1. Upon comparison of the patient characteristics and outcomes between early and delayed initiation groups, neoadjuvant therapy, American Society of Anesthesiologists Physical Status (ASA‐PS), cardiovascular disease, operation time, and postoperative complications were significantly associated with the initiation timing of adjuvant therapy.

**TABLE 1:** Comparison of the patient characteristics and outcomes between early and delayed initiation groups (*n* = 444).

Variables	Initiation timing of adjuvant therapy		*p*‐Value
	Early, *n* = 328 (%)	Delayed, *n* = 116 (%)	
Patient characteristics			
Age, ≥80 years	34 (10)	17 (15)	0.223
Sex, female	155 (47)	48 (41)	0.274
Resectability status, BR/LA	110 (34)	40 (34)	0.853
Neoadjuvant therapy	115 (35)	54 (47)	0.030
ASA‐PS, ≥3	5 (2)	8 (7)	0.006
BMI, ≥25	48 (15)	22 (19)	0.279
Cardiovascular disease	24 (7)	17 (15)	0.025
Pulmonary disease	18 (5)	11 (9)	0.149
Cancer history	57 (17)	18 (16)	0.643
Diabetes	112 (34)	51 (44)	0.061
Biliary drainage	111 (34)	41 (35)	0.770
PNI, <40	131 (40)	46 (40)	0.957
Preoperative CA19‐9, ≥37 U/mL	167 (51)	65 (56)	0.342
Operative factors			
Procedure, PD	211 (64)	76 (66)	0.818
Portal vein resection	92 (28)	37 (32)	0.435
Arterial resection	39 (12)	19 (16)	0.227
Operation time, ≥400 min	71 (22)	37 (32)	0.034
Estimated Blood loss, ≥1000 mL	81 (25)	31 (27)	0.703
Transfusion	43 (13)	20 (17)	0.281
Postoperative complication, ≥III	18 (5)	39 (34)	<0.001
Pathological findings			
Pathological tumor size, ≥30 mm	177 (54)	66 (57)	0.585
Tumor differentiation, grade2/3	218 (66)	71 (61)	0.310
Lymphovascular invasion, positive	190 (58)	76 (66)	0.149
Perineural invasion, positive	261 (80)	99 (85)	0.164
pT, T3/4	64 (20)	29 (25)	0.218
pN, positive	197 (60)	76 (66)	0.297
Surgical margin, positive	58 (18)	20 (17)	0.914

Abbreviations: ASA‐PS, American Society of Anesthesiologists Physical Status Classification; BMI, body mass index; BR, borderline resectable; CA19‐9, Carbohydrate antigen 19–9; LA, locally advanced; PD, pancreatoduodenectomy; PNI, prognostic nutritional index.

### 3.2 Survival analysis

**FIGURE 2:**
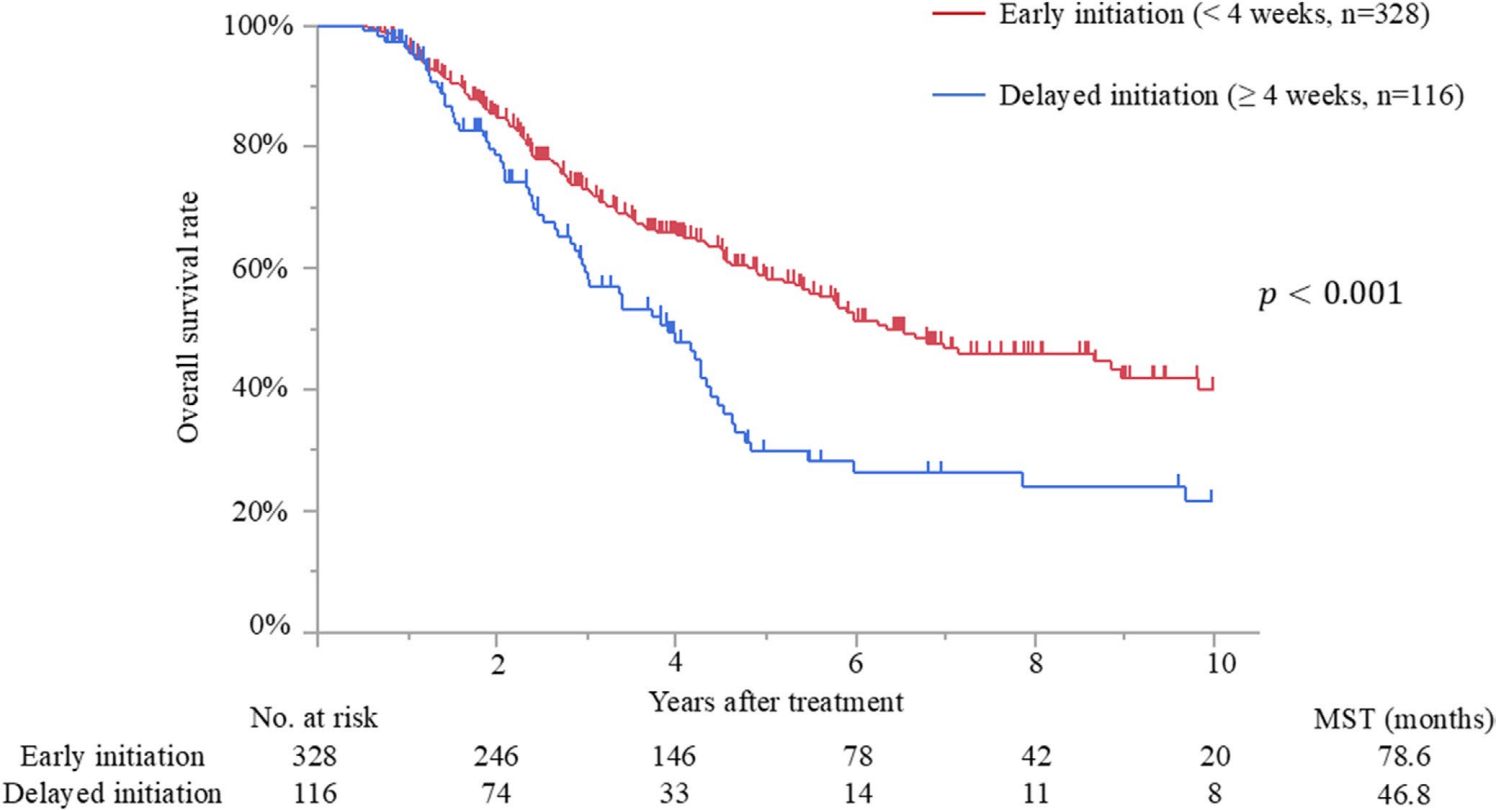
Overall survival curves of patients with early or delayed adjuvant initiation. MST, median survival time.

**FIGURE 3:**
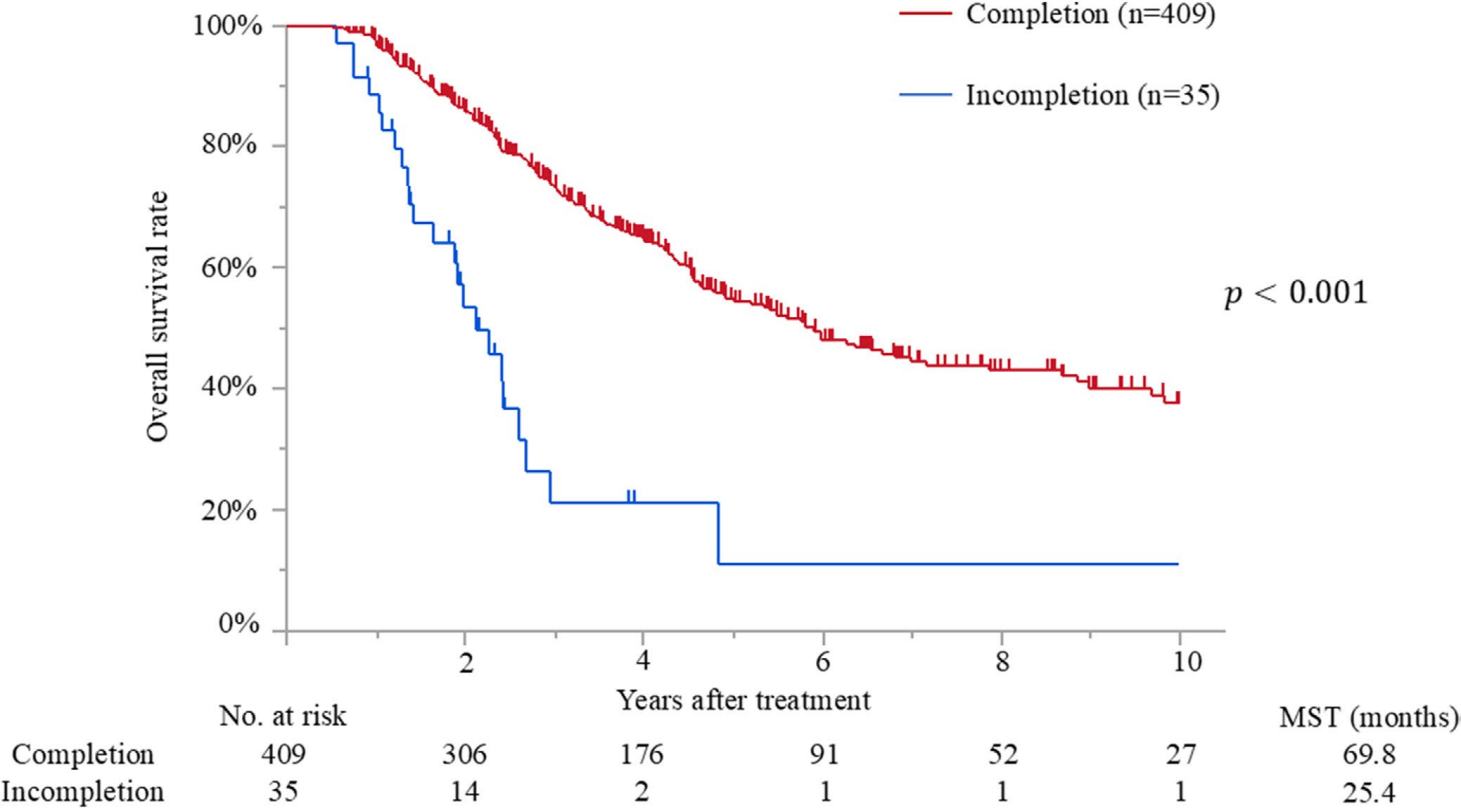
Overall survival curves of patients with adjuvant completion or incompletion. MST, median survival time.

**FIGURE 4:**
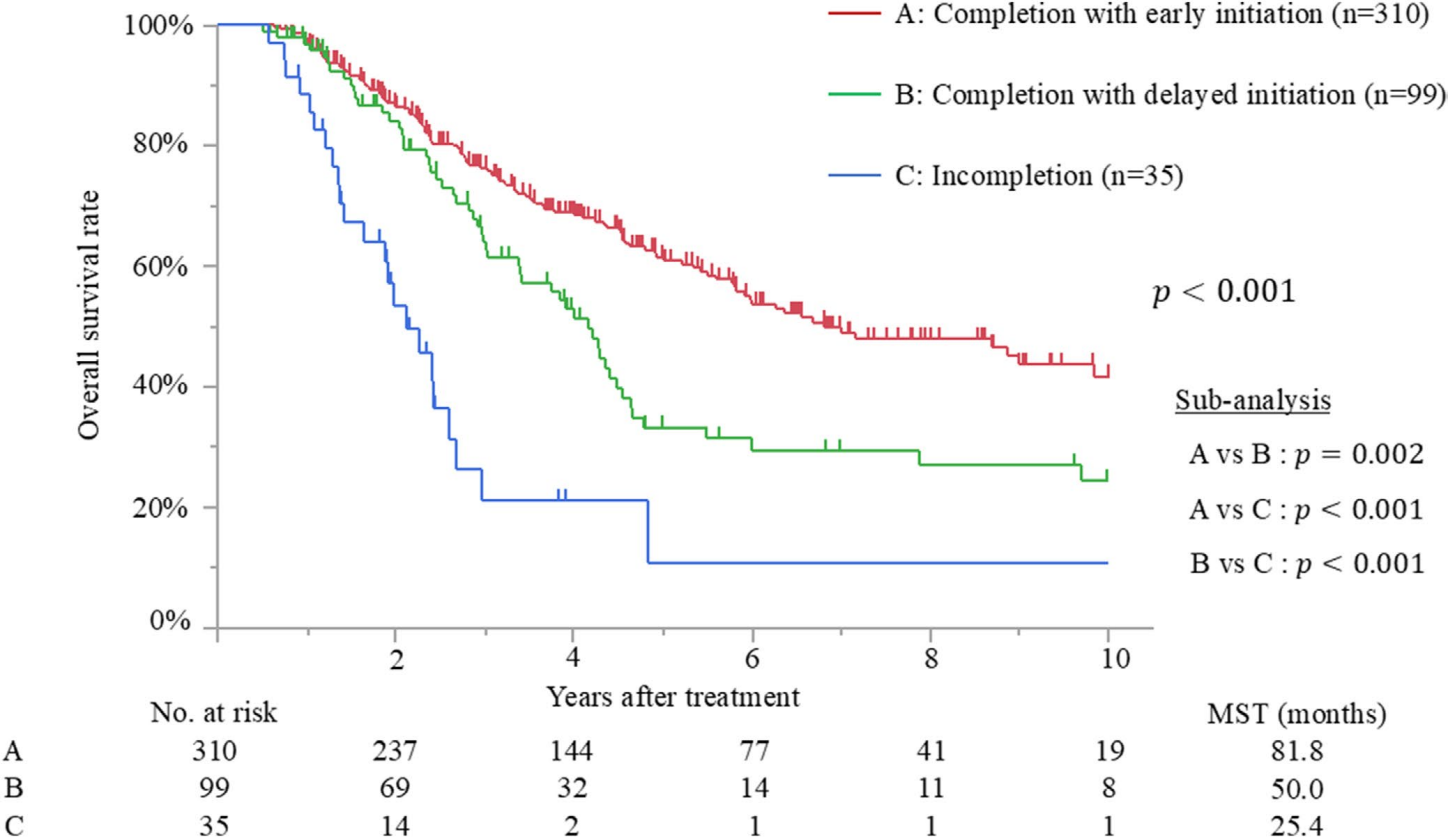
Overall survival curves among patients with adjuvant completion with early or delayed initiation and incompletion. MST, median survival time.

The median follow‐up time after surgery for the censored patients was 47.4 months (IQR, 25.2–81.7 months). Median survival and 5‐year overall survival rate were 65.0 months and 52%, respectively. Patients with early initiation of adjuvant therapy had better overall survival than those with delayed initiation (median survival time [MST], 78.6 months vs. 46.8 months; *p* < 0.001) (Figure 2). The MSTs of patients who initiated adjuvant treatment <2, 2–4, 4–6, 6–8, and >8 weeks after surgery were 140.7, 76.4, 46.8, 40.4, and 52.7 months, respectively (*p* = 0.009). Moreover, patients who completed adjuvant therapy had better survival than those who did not (MST, 69.8 months vs. 25.4 months; *p* < 0.001) (Figure 3). Recurrence was detected in 228 patients (51%), and the most frequent recurrence site was local (*n* = 76), followed by lung (*n* = 62), liver (*n* = 53), peritoneum (*n* = 28), and others (*n* = 9). Multivariate analysis demonstrated that biliary drainage, elevated preoperative CA19‐9, pT3/4 status, nodal positive, and surgical margin positive were independent poor prognostic factors, while adjuvant early initiation (HR, 0.66; *p* = 0.009) and completion (HR, 0.35; *p* < 0.001) were independent prognostic factors for prolonged survival (Table 2). When the 444 patients were divided into three groups according to adjuvant completion and initiation status: (A) adjuvant completion with early initiation (*n* = 310), (B) adjuvant completion with delayed initiation (*n* = 99), and (C) adjuvant incompletion (*n* = 35), patients who experienced adjuvant completion with early initiation had better survival than those who experienced adjuvant completion with delayed initiation (*p* = 0.002) and those with adjuvant incompletion (*p* < 0.001), whereas those who experienced adjuvant completion with delayed initiation had better survival than those with adjuvant incompletion (*p* < 0.001). These survival curves are shown in Figure 4.

**TABLE 2:** Survival analysis of PDAC patients with adjuvant therapy initiation (*n* = 444).

Variables	Univariate			Multivariate		
	No. of patients (%)	MST (months)	*p*‐Value	HR	95% CI	*p*‐Value
Age, ≥80 years	51 (11)	86.0	0.868			
Sex, female	203 (46)	59.0	0.450			
Resectability status, BR/LA	150 (34)	46.8	0.005	1.18	0.83–1.67	0.362
Neoadjuvant therapy	169 (38)	58.0	0.838			
ASA‐PS, ≥3	13 (3)	40.3	0.047	1.37	0.62–3.03	0.435
BMI, ≥25	70 (16)	64.1	0.807			
Cardiovascular disease	41 (9)	58.0	0.375			
Pulmonary disease	29 (7)	46.1	0.050			
Cancer history	75 (17)	71.8	0.739			
Diabetes	163 (37)	54.1	0.029	1.25	0.94–1.68	0.129
Biliary drainage	152 (34)	49.1	<0.001	1.60	1.17–2.18	0.003
PNI, <40	177 (40)	57.7	0.120			
Preoperative CA19‐9, ≥37 U/mL	232 (52)	54.4	<0.001	1.43	1.06–1.92	0.019
Procedure, PD	287 (65)	57.7	0.142			
Portal vein resection	129 (29)	41.5	<0.001	1.23	0.84–1.80	0.282
Arterial resection	58 (13)	48.1	0.267			
Operation time, ≥400 min	108 (24)	38.6	0.004	1.13	0.79–1.61	0.514
Estimated blood loss, ≥1000 mL	112 (25)	42.1	<0.001	1.07	0.75–1.55	0.699
Transfusion	63 (14)	32.6	<0.001	1.35	0.89–2.04	0.151
Postoperative complication, ≥III	57 (13)	44.9	0.315			
pT3/4	93 (21)	35.0	<0.001	1.72	1.22–2.42	0.002
pN, positive	273 (61)	44.9	<0.001	2.34	1.64–3.35	<0.001
Surgical margin, positive	78 (18)	32.6	<0.001	1.79	1.28–2.51	<0.001
Adjuvant initiation, early (<4 weeks)	328 (74)	78.6	<0.001	0.66	0.48–0.90	0.009
Adjuvant completion, complete	409 (92)	69.8	<0.001	0.35	0.21–0.57	<0.001

Abbreviations: 95% CI, 95% confidence interval; ASA‐PS, American Society of Anesthesiologists Physical Status Classification; BMI, body mass index; BR, borderline resectable; CA19‐9, carbohydrate antigen 19–9; HR, hazard ratio; LA, locally advanced; MST, median survival time; PD, pancreatoduodenectomy; PDAC, pancreatic ductal adenocarcinoma; PNI, prognostic nutritional index.

With respect to the relative dose intensity (RDI) of adjuvant therapy, when patients were divided into high (≥50%, *n* = 383) and low (<50%, *n* = 61) RDI groups, patients who received high RDI had significantly better survival than those who received low RDI (MST, 69.7 months vs. 38.7 months; *p* < 0.001). In comparisons of survival outcomes between adjuvant GS and S‐1 therapies, the MSTs of patients with GS and S‐1 therapies were 65.9 and 71.8 months, respectively (*p* = 0.291).

Evaluation of the optimal cut‐off timing of adjuvant initiation is demonstrated in Table 3. The MSTs between early and delayed adjuvant initiation were compared, with cut‐off timings of adjuvant therapy set from 2 to 10 weeks after surgery. Early groups had significantly better survival than delayed groups when the cut‐off timing was 3, 4, 5, and 7 weeks, whereas no significant differences were found between these groups when the cut‐off timing was 2, 6, 8, 9, and 10 weeks.

**TABLE 3:** Evaluation of optimal cut‐off timing of adjuvant initiation.

Cut‐off timing	MST (months)		*p*‐Value
	Early	Delayed	
2 weeks (Early, *n* = 43/Delayed, *n* = 401)	140.7	62.5	0.131
3 weeks (Early, *n* = 245/Delayed, *n* = 199)	78.6	51.4	0.006
4 weeks (Early, *n* = 328/Delayed, *n* = 116)	78.6	46.8	<0.001
5 weeks (Early, *n* = 382/Delayed, *n* = 62)	69.7	40.7	0.022
6 weeks (Early, *n* = 412/Delayed, *n* = 32)	69.1	44.9	0.073
7 weeks (Early, *n* = 418/Delayed, *n* = 26)	69.1	44.9	0.015
8 weeks (Early, *n* = 424/Delayed, *n* = 20)	65.9	52.7	0.166
9 weeks (Early, *n* = 429/Delayed, *n* = 15)	65.9	52.7	0.404
10 weeks (Early, *n* = 432/Delayed, *n* = 12)	65.8	53.7	0.323

Abbreviation: MST, median survival time.

The analysis of risk factors preventing adjuvant completion with early initiation is shown in Table 4. The multivariate analysis revealed that ASA‐PS ≥3 (OR, 4.64; *p* = 0.024), cardiovascular disease (OR, 2.18; *p* = 0.036), postoperative complications ≥III (OR, 5.88; *p* < 0.001), and pT3/4 (OR, 1.77; *p* = 0.036) were independent risk factors of adjuvant incompletion with early initiation.

**TABLE 4:** Risk factors preventing adjuvant completion with early initiation (*n* = 444).

Variables	Univariate			Multivariate	
	No. of patients (%)	OR (95% CI)	*p*‐Value	OR (95% CI)	*p*‐Value
Age, ≥80 years	51 (11)	1.73 (0.95–3.16)	0.076		
Sex, female	203 (46)	1.15 (0.77–1.73)	0.498		
Resectability status, BR/LA	150 (34)	1.31 (0.86–2.00)	0.213		
Neoadjuvant therapy	169 (38)	1.71 (1.13–2.58)	0.011	1.40 (0.84–2.32)	0.194
ASA‐PS, ≥3	13 (3)	5.51 (1.67–18.2)	0.003	4.64 (1.23–17.5)	0.024
BMI, ≥25	70 (16)	1.35 (0.79–2.31)	0.278		
Cardiovascular disease	41 (9)	2.69 (1.41–5.16)	0.003	2.18 (1.05–4.51)	0.036
Pulmonary disease	29 (7)	2.29 (1.07–4.90)	0.035	1.64 (0.70–3.85)	0.252
Cancer history	75 (17)	1.03 (0.60–1.76)	0.920		
Diabetes	163 (37)	1.36 (0.90–2.06)	0.146		
Biliary drainage	152 (34)	0.96 (0.63–1.47)	0.849		
PNI, <40	177 (40)	1.11 (0.74–1.69)	0.609		
Preoperative CA19‐9, ≥37 U/mL	232 (52)	1.29 (0.86–1.94)	0.215		
Procedure, PD	287 (65)	1.01 (0.67–1.56)	0.934		
Portal vein resection	129 (29)	1.23 (0.79–1.91)	0.357		
Arterial resection	58 (13)	1.93 (1.09–3.39)	0.025	1.32 (0.66–2.64)	0.428
Operation time, ≥400 min	108 (24)	1.67 (1.06–2.63)	0.029	1.38 (0.83–2.29)	0.209
Estimated blood loss, ≥1000 mL	112 (25)	1.25 (0.79–1.97)	0.348		
Transfusion	63 (14)	1.52 (0.87–2.64)	0.147		
Postoperative complication, ≥III	57 (13)	6.66 (3.64–12.2)	<0.001	5.88 (3.12–11.0)	<0.001
pT, T3/4	93 (21)	1.73 (1.07–2.79)	0.026	1.77 (1.04–3.01)	0.036
pN, positive	273 (61)	1.23 (0.81–1.88)	0.326		
Surgical margin, positive	78 (18)	1.03 (0.61–1.76)	0.901		

Abbreviations: ASA‐PS, American Society of Anesthesiologists Physical Status Classification; BMI, body mass index; BR, borderline resectable; CA19‐9, carbohydrate antigen 19–9; LA, locally advanced; PD, pancreatoduodenectomy; PNI, prognostic nutritional index.

## 4 DISCUSSION

Adjuvant therapy is the most important treatment strategy for improving the survival of patients with curatively resected PDAC. Several survival analyses^2^, ^3^ of patients with PDAC have revealed that adjuvant therapy is strongly associated with prolonged survival, as well as tumor markers.^17^, ^18^ However, the optimal timing of adjuvant initiation remains unclear. During the study period, the treatment policy in which recovered patients received adjuvant therapy as early as possible was consistent. Consequently, the median time to initiation of adjuvant therapy was 20 days, and 74% of eligible patients received adjuvant therapy within 4 weeks after surgery. Moreover, the adjuvant completion rate in this study was relatively high (92%), probably because patients in good condition intended to complete adjuvant therapy. In this study, patients with early recurrence or without adjuvant initiation were excluded, and the results of this study reflect the survival effects of adjuvant therapy in patients with resected PDAC.

Survival analysis revealed that both early initiation and completion of adjuvant therapy were independent prognostic factors. The MST of the adjuvant completion with early initiation group was 81.8 months, a relatively better prognosis than that noted in previous studies, which had MSTs of 22–57 months.^4^, ^5^, ^6^, ^7^, ^8^ Valle et al.^9^ demonstrated that six cycles of adjuvant completion rather than early initiation contributed to prolonged survival. Other studies have focused not on adjuvant completion but on adjuvant timing.^4^, ^5^, ^6^, ^7^, ^8^ Early initiation was better than delayed initiation in some studies,^4^, ^5^ whereas it was equivalent in other studies.^6^, ^7^, ^8^ This study set the cut‐off timing of adjuvant initiation at 4 weeks, relatively earlier than those of previous studies, and found that the early initiation group had better survival than the delayed initiation group. In addition, when the cut‐off timings of adjuvant initiation were set at 3, 4, 5, and 7 weeks, the early groups had prolonged survival, whereas there were no significant differences when the cut‐off timings were set at 2, 6, 8, 9, and 10 weeks. Some studies^6^, ^7^, ^8^ without significant differences between the early and delayed groups set the cut‐off timing at 9–12 weeks. These results suggest that the results may differ depending on the cut‐off timing between early and delayed initiation.

Interestingly, this study revealed that the adjuvant completion with delayed initiation group had a significantly better prognosis than the incompletion group, but still had a significantly poorer prognosis than the early initiation group. With respect to the recurrence patterns in this study, which excluded patients without adjuvant therapy or early recurrence within 6 months after surgery, 143 (63%) patients had distant metastases in the liver, lung, and peritoneum. Delayed initiation of adjuvant therapy due to postoperative inflammation, immunosuppression, or complications may cause the progression of distant occult metastases.^19^, ^20^ From the viewpoint of basic science, Javed et al.^21^ demonstrated that postoperative circulating tumor cell positivity was associated with poor survival in patients who either experienced delayed initiation or lack of adjuvant therapy. They hypothesized that a delay or lack of adjuvant therapy allowed circulating tumor cells to proliferate and could potentially cause residual systemic disease, subsequently resulting in disease recurrence. In addition, this study suggested that it was insufficient to complete adjuvant therapy, even if delayed, but rather that adjuvant completion with early initiation led to prolonged survival.

The risk factors for delayed or incomplete adjuvant therapy were poor patient status, such as a higher ASA‐PS or combined cardiovascular disease, and severe postoperative complications. An American nationwide survey^22^ revealed that postoperative complications were common following pancreatic surgery and were associated with the omission of adjuvant chemotherapy and treatment delays. Postoperative complications following pancreatic surgery mainly include surgical site infections, such as postoperative pancreatic fistula, abdominal abscess, and anastomotic leakage. Postoperative infections can cause immunodeficiency and delayed recovery. To administer cytotoxic chemotherapy agents to patients after surgery, sufficient recovery and good general condition are required. A multicenter cohort study by the Japanese Society of Surgical Infection^23^ reported that postoperative infection after pancreatic cancer surgery was strongly associated with adjuvant incompletion and indirectly caused a worse prognosis. In addition, this study demonstrated that postoperative complications were strongly associated with delayed initiation or incomplete adjuvant therapy. Careful perioperative management and surgical skills to prevent postoperative complications are expected to improve the survival of patients with PDAC.

This study has some limitations. First, this was a retrospective cohort study with a relatively small number of patients with PDAC at a single institution. There were several potential biases in patient selection, neoadjuvant strategies, surgical skills, and follow‐up strategies over the long study period. However, early adjuvant strategies remained consistent throughout the study period. To overcome these limitations, prospective studies with a larger number of patients with resected PDAC who intend to receive early adjuvant therapy after surgery at multiple institutions are warranted. Second, to prove the survival benefit of early adjuvant initiation, comparisons of the prognostic significance between early and delayed adjuvant therapy in patients with good status and good postoperative course are required. A randomized control trial is warranted. Finally, the adjuvant therapy in this study included only chemotherapy consisting of S‐1 and/or gemcitabine. The timing and duration of adjuvant radiation and chemoradiotherapy were not considered. Moreover, the survival benefits of stronger combined regimens^24^, ^25^, ^26^ for high‐risk patients and precision medicine, including gene or immunotherapy,^27^, ^28^, ^29^ should be considered adjuvant strategies in the future.

In conclusion, adjuvant completion with early initiation may contribute to better survival in patients with PDAC, and the prevention of severe postoperative complications may facilitate adjuvant completion with early initiation.

## AUTHOR CONTRIBUTIONS

**Kenjiro Okada:** Conceptualization; data curation; formal analysis; investigation; methodology; project administration; writing – original draft. **Kenichiro Uemura:** Conceptualization; methodology; project administration; supervision; writing – review and editing. **Tatsuaki Sumiyoshi:** Conceptualization; data curation; writing – review and editing. **Ryuta Shintakuya:** Conceptualization; data curation; writing – review and editing. **Kenta Baba:** Data curation; writing – review and editing. **Takumi Harada:** Data curation; writing – review and editing. **Yasutaka Ishii:** Conceptualization; writing – review and editing. **Shiro Oka:** Conceptualization; writing – review and editing. **Yoshiaki Murakami:** Conceptualization; writing – review and editing. **Shinya Takahashi:** Conceptualization; project administration; writing – review and editing.

## FUNDING INFORMATION

The authors received no financial support for the research, authorship, and/or publication of this article.

## CONFLICT OF INTEREST STATEMENT

The authors declare no conflict of interests for this article.

## ETHICS STATEMENT

Approval of the research protocol by an Institutional Reviewer Board: The protocol for this research project has been approved by the Ethics Committee of Hiroshima University (Approval No. E‐2151) and it conforms to the provisions of the Declaration of Helsinki.

Informed Consent: N/A.

Registry and the Registration No. of the study/trial: N/A.

Animal Studies: N/A.

## Supporting information


Table S1.
