# Supplementary material for: Survival benefit of adjuvant therapy completion with early initiation for patients with pancreatic ductal adenocarcinoma
Source: Ann Gastroenterol Surg. 2024 Dec 25;9(4):785–93. doi: 10.1002/ags3.12898 (PMC12211095; doi:10.1002/ags3.12898)
Supplement: Supplementary file 1 — Table S1. [file AGS3-9-785-s001.docx]

**Supporting information. Pretreatment, surgical and pathological factors (n=444).**

|  | No. of patients (%) |
| --- | --- |
| **Pretreatment factors** |  |
| Pretreatment tumor size, median | 25 (IQR, 19-32) mm |
| cN status | cN0 257 (58), cN1 187 (42) |
| Neoadjuvant therapy | 169 (38) |
| Neoadjuvant regimens |  |
| Gemcitabine+S-1 | 60 (36) |
| Gemcitabine+nab-PTX | 16 (9) |
| Gemcitabine+nab-PTX+S-1 | 80 (47) |
| Modified FOLFIRINOX | 13 (8) |
| Radiation | 0 |
| Post-neoadjuvant tumor size, median | 23 (IQR, 15-30) mm |
| RECIST | PR 55 (33), SD 114 (67) |
| **Surgical outcomes** |  |
| CR-POPF | 50 (11) |
| DGE | 9 (2) |
| Hemorrhage | 6 (1) |
| Reoperation | 6 (1) |
| **Pathological factors** |  |
| Lymphatic invasion | 266 (60) |
| Venous invasion | 202 (45) |
| Perineural invasion | 360 (81) |
| Histological response (neoadjuvant therapy) | Major 24 (14), Complete 3 (2) |

IQR, interquartile range; cN status, clinical nodal status; nab-PTX, nab-paclitaxel, FOLFIRINOX, leucovorin, fluorouracil, irinotecan, and oxaliplatin; RECIST, response evaluation criteria in solid tumors; PR, partial response; SD, stable disease; CR-POPF, clinically relevant postoperative pancreatic fistula; DGE, delayed gastric emptying
